# Effects of Feeding Increasing Levels of Yerba Mate on Lamb Meat Quality and Antioxidant Activity

**DOI:** 10.3390/ani10091458

**Published:** 2020-08-20

**Authors:** Yuli A. Pena-Bermudez, Richard R. Lobo, Danny A. Rojas-Moreno, Mirele D. Poleti, Tamyres R. de Amorim, Alessandra F. Rosa, Angélica S. C. Pereira, Rafael S. B. Pinheiro, Ives C. S. Bueno

**Affiliations:** 1 Department of Animal Science, College of Animal Science and Food Engineering, University of São Paulo/FZEA, Pirassununga 13635-900, São Paulo, Brazil; richardrobertolobo@hotmail.com (R.R.L.); dannymoreno.zoot@gmail.com (D.A.R.-M.); tamyamorim@usp.br (T.R.d.A.); ivesbueno@usp.br (I.C.S.B.); 2 Department of Veterinary Medicine, College of Animal Science and Food Engineering, University of São Paulo (USP), Pirassununga 13635-900, São Paulo, Brazil; mdpoleti@gmail.com (M.D.P.); afrosa@usp.br (A.F.R.); 3 Department of Nutrition and Animal Production, College of Veterinary Medicine and Animal Science, University of São Paulo (USP), Pirassununga 13635-900, São Paulo, Brazil; angelpereira@usp.br; 4 Department of Biology and Animal Science, Faculty of Engineering, State University of São Paulo (UNESP), Ilha Solteira 13635-900, São Paulo, Brazil; rafael.pinheiro@unesp.br

**Keywords:** oxidative stability, natural antioxidants, useful life, meat quality, polyphenolic

## Abstract

**Simple Summary:**

The population’s growing concern for health and the increased consumption of natural products have led to the study of the use of bioactive compounds in animal feed, especially those containing antioxidants. An example of this is yerba mate (*Ilex paraguariensis*), a plant highly consumed in South America due to its antioxidant properties, which benefit human health and can reduce the incidence of cardiovascular diseases. However, the effects of yerba mate on animal feed as well as the composition and preservation of meat products are unknown. Therefore, the objective of the current study was to evaluate the effects of the inclusion level of yerba mate extract in the lamb’s diet on meat quality traits, antioxidant activity, and shelf-life. Our results showed that the use of the extract in lamb feed did not negatively affect the characteristics of lamb meat nor increase lipoperoxidation during a six-day storage period. The inclusion of 4% yerba mate extract (YME) resulted in a higher value in the yellow colour of the meat.

**Abstract:**

The present study investigated the inclusion of yerba mate extract (YME) in the lamb’s diet on meat quality traits, antioxidant activity, and shelf-life. Thirty–six lambs were distributed according to a block design with the following groups: control group without YME (0%) and three treatment groups with 1, 2, and 4% YME inclusion in the dry matter. The animals were fed these diets for 53 days. Samples were collected from the *Longissimus* *thoracis* (LT) muscle to analyze antioxidant activity and meat quality. Samples were placed on a counter display simulating a retail environment for 0, 3, and 6 days at 4 ± 2 °C. All data were analyzed using a MIXED model with orthogonal contrasts. Inclusion of 1 and 4% YME in the diet changed the yellow (b*) and the chroma (C*) of the meat (*p* ≤ 0.05). The pH, colour, thiobarbituric acid reactive substances, and carbonyl values were influenced by the retail display time for all the evaluated treatments (*p* ≤ 0.03). However, neither diet nor the retail display time influenced the oxidation of proteins or the antioxidant enzyme activities of catalase (CAT), superoxide dismutase (SOD), glutathione peroxidase (GPX) and glutathione activity (GSH) in meat. Therefore, the inclusion of 4% YME showed positive results in the yellow and colour stability parameters of the meat without increasing the lipid peroxidation values or altering the normal meat quality parameters in lambs.

## 1. Introduction

Meat is considered a food with high nutritional quality and is of vital importance due to its functional characteristics and rich source of proteins, zinc, and B vitamins [1]. Accordingly, maintaining quality in animal production is of great importance to obtain a final product that satisfies the consumer’s demand for appearance and composition [2]. Because meat is strongly influenced during the storage process by microbiological deterioration and oxidation of its compounds, conservation of meat is important [3]. The final products of these reactions can lead to changes in composition, leading to market rejection and loss of profit or economic loss [4].

In the preservation of food products, antioxidant substances from a synthetic or natural origin are used to slow down the oxidative process by removing pro-oxidants, such as free fatty acids, metals, and oxidized compounds, and protecting food from light [5]. However, there is a crucial need for regulatory monitoring of synthetic antioxidants in animal feed, considering their potential toxicity in human health [6,7,8,9]. Due to the food safety concerns of consumers, research on the use of natural antioxidants, mainly active substances present in plants that include tannins, saponins, essential oils, and phenolic compounds, has increased [10,11]. These compounds can be used directly or in combination with other animal feed components, delaying the oxidative process and contributing to animal welfare and antioxidant status [12,13,14,15,16,17]. 

In this context, yerba mate (*Ilex paraguariensis* St. Hilaire), a native plant grown in South American Countries and widely consumed in these regions [18], has been used for its antioxidant properties. This plant is composed mainly of polyphenolic compounds (chlorogenic acid) and xanthines (caffeine and theobromine) as well as purine alkaloids, flavonoids and saponins in smaller amounts [19,20]. The use of yerba mate for human health is beneficial, providing a lower incidence of cardiovascular disease and cancer due to the presence of phenolic compounds, mainly chlorogenic acids [10,21]. These phenolic compounds have the ability to scavenge free radicals, inhibiting lipid peroxidation when used in food preservation [19]. In animal production, previous studies showed that Yerba Mate improved lactation performance (increased milk fat, protein, and total solids content) of ewes and the growth rate of lambs [22,23]. Supplementation of yerba mate for large and small ruminants resulted in lower oxidative stress and also improved their productive and reproductive performance [24,25]. Finally, in meat quality in beef cattle and poultry, it was shown to be an additive that improves oxidative stability, nutritive value, and sensory quality [26,27,28].

To the best of our knowledge, limited information on yerba mate and its implication on meat quality is available [28,29,30], thus requiring more studies to better understand the mechanisms of action of yerba mate. We hypothesized that the inclusion of YME in the diet could positively alter the antioxidant status of the muscle during the retail display time and consequently, the meat quality parameters (color, oxidation of lipids, and proteins) of lamb. This study aimed to investigate the effect of inclusion levels of YME in the diet of lambs on meat quality traits, antioxidant activity, and shelf-life.

## 2. Materials and Methods

The experiments were performed in the Department of Animal Science, College of Animal Science and Food Engineering (FZEA), University of São Paulo (USP), Pirassununga, São Paulo, Brazil (21°57′02″ S, 47°27′50″ W). The Animal Use Ethics Commission (CEUA) of FZEA/USP, (Protocol number CEUA 3497040618) approved all procedures using animals.

### 2.1. Diets, Experimental Design, and Animal Management

Thirty-six male uncastrated industrial crossbred lambs [(Texel × Santa Inês) × Dorper] with an average initial weight of 23.90 ± 3.67 kg and approximately 90 days of age were used. All animals were treated for internal and external parasites using a commercial drug with levamisole hydrochloride as the active ingredient, following the manufacturer’s dose recommendations. The experiment was carried out in a shed (width = 5 m, length = 12 m and height = 4 m) in which the lambs were housed in individual pens (1 m ^2^) with a raised plastic floor (50 cm), allowing the passage of feces and urine to the concrete floor; each pen had individual access to water and feeder.

The animals were divided into nine blocks, and each block had four animals with similar initial weight. The four animals in each block were randomly assigned into one of the four treatments, following a randomized block design where each treatment had nine animals. Dietary treatments included no inclusion of YME (0%) and three treatments with 1, 2, and 4% YME of inclusion on a dry matter basis (DM), using food-grade kaolin as an inert ingredient. All animals received a control diet for three days, and on the fourth day, the animals were fed the predetermined diets for a 50-day growth trial.

The feed was formulated based on the National Research Council’s [31] requirements. The forage:concentrate ratio was 40:60 with corn silage (*Zea mays*) and grain concentrate, which included ground corn, soybean meal, and a mineral mixture for sheep (Table 1). The diet was offered twice a day at 08:00 h and 16:00 h, and water was offered ad libitum. The feed provided and orts were weighed daily to determine the daily DM intake, allowing 10% orts. The animal performance was described in a companion paper [32].

### 2.2. Chemical Analysis

Diet ingredients were analyzed by the Association of Official Analytical Chemists [33] methods for dry matter (DM, method 934.01), ash (ASH, method 923.03), ether extract (EE, method 920.85), and crude fiber (CF, method 978.10). Crude protein (CP, method 920.87) was analyzed by the Kjeldahl method. Neutral detergent fiber (NDF) was analyzed according to Mertens [34] using the amylase enzyme method, and acid detergent fiber (ADF) and lignin (LN) were measured according to method 973.18 by Van Soest et al. (1991). Gross energy (GE) was measured with a calorimetric pump (C200 System, IKA, Staufen, Germany). Organic matter (OM) was calculated by subtracting the ASH content from 100. These values are shown in Table 1.

The YME was produced by Centro Flora (Botucatu, São Paulo, Brazil) from fresh leaves of *Ilex paraguariensis* via water:ethanol 75:25 *v*/*v* extraction at 90 °C, and the total phenolic concentration was 21.7 eq–g gallic acid/100 g extract and 6% caffeine (*w*/*w*). The extract given to the animals in this research was the same product previously used by Zawadzki et al. [28]. In this extract, a complete characterization of phenolic compounds was reported using an ionization mass spectrometry analysis by electrospray ultra-performance liquid chromatography, within most relevant phenolic compounds in the YME chemical composition, which were as follows (expressed in mg g^−1^): chlorogenic acid, 12.30 ± 0.01; 1,5-dicaffeoylquinic acid, 6.01 ± 0.01; caffeic acid, 813 ± 0.002; ferulic acid, 0.0545 ± 0.0008; gallic acid, 0.018 ± 0.004. The extract was mixed with the concentrate, and the concentrate was mixed into the silage at feeding time. The chemical composition of the experimental diets and YME were presented and discussed in a companion paper [32].

### 2.3. Slaughter Procedures and Meat Sampling

The lambs were kept in confinement for 53 days and slaughtered with an approximate body weight of 40 kg [32] in the slaughterhouse at the, College of Animal Science and Food Engineering (FZEA), University of São Paulo (USP) after solid fasting for 16 h and receiving only water ad libitum. The slaughtering procedures were performed according to the regulation of the industrial and sanitary inspection of animal products of the Ministry of Agriculture, Livestock, and Supply [35]. *Longissimus thoracis* (LT) muscle samples (12 g) were collected 45 min after slaughter using a biopsy punch, packed in aluminum foil and frozen in liquid nitrogen for the analysis of antioxidant enzymes including catalase (CAT), superoxide dismutase (SOD), glutathione peroxidase (GPX) and reduced glutathione (GSH). The average hot carcass weight was 17 kg [32]. The carcasses were chilled at 2 °C for 24 h, after which they were divided longitudinally, and the right LT muscle (between 10 and 12 ribs) was extracted for meat quality analysis. More detail about carcass metrics and yield were described in a companion paper [32]

### 2.4. Meat Analysis

After removing the LT from the carcass, it was divided into 6 slices. Three slices were used for the analysis of lipid oxidation (each 1.5 cm thickness) and the other three were used to assess protein oxidation (1.5 cm thickness). The slices were packed in polystyrene trays and after covered with plastic wrap. Then they placed in a commercial display refrigerator (Auden Model Vega 125 Lx C) with lighting (Philips TL-D 58 W/33-640 1SL/25) on the side columns for a 0, 3 and 6-day shelf-life study at 4 ± 2 °C simulating retail display conditions, the trays were not rotated during the evaluation. pH, instrumental colour, lipid, and protein oxidation of meat was conducted for all three-time periods across all treatments (i.e., 0, 1, 2, and 4% YME). On sampling days, the slices were unpacked, subjected to the analysis of the pH and colour, subsequently wrapped in aluminum foil, and stored at −20 °C for further analysis.

#### 2.4.1. pH Measurement and Meat Colour Determination

Sample pH was determined using a portable pH meter with a penetration probe (Model HI 99163, Hanna^®^ Mark). Measurements were performed on samples immediately after slices were removed from packages after exposure for 0, 3, and 6 days.

Instrumental surface coluor analysis was performed using a colorimeter (Miniscan EZ, Hunter Lab brand) with a D65 light source, a 10° observation angle, and a cell opening measuring 30 mm. The CIELAB system was used to determine the following colour components: L* (brightness, 0 = black, 100 = white), a* (green (−100) to red (+100)) and b* (blue (−100) to yellow (+100)) [36]. The slope angle (*H*°, expressed in degrees) and chroma (C*) levels were also calculated as

(1) $$H{}^{\circ}=\tan^{-1}\left(b^{*}/a^{*}\right)\times \;\left(180/\pi \right)$$

and

(2) $$C^{*}=\sqrt{a^{2}}+b^{2}$$

respectively. Colour measurements were recorded at 0, 3, and 6 days of display. 

#### 2.4.2. Analysis of Lipid and Protein Oxidation

The analysis of thiobarbituric acid reactive substances (TBARS) was performed following the methodology proposed by Sorensen and Jørgensen [37]. Approximately 5 g of each sample of LT muscle was homogenized in a Turratec homogenizer (Model T10 basic, IKA brand) using 15 mL of TCA solution (7.5% trichloroacetic acid, 0.1% EDTA disodium salt, and 0.1% n-propylgalate). Absorbance readings for samples and blanks were performed on a spectrophotometer (Multiskan Go, Thermo Scientific Brand) at 530 nm and 600 nm. A standard curve with 5 points was prepared using a solution of tetraethoxypropane of known concentration. The malonaldehyde (MDA) concentration of the samples was obtained with the equation provided by the standard curve. The analyses were performed in duplicate and expressed in mg of MDA per kg of meat.

Protein oxidation was determined using a carbonyl assay kit (code: DCAR-100; BioAssay Systems, Hayward, CA, USA) and an improved method, in which 2,4-dinitrophenylhydrazine (DNPH) reacts with carbonyl groups to produce a coloured compound at 375 nm. The intensity of this coloured compound is directly proportional to the carbonyl content present in the sample, and the values are presented in nmol/mg of protein [38].

#### 2.4.3. Antioxidant Enzyme Activity

Enzyme analyses were measured from meat and determined using kits from BioAssay Systems (Hayward, CA, USA) according to the methodology proposed in each extraction protocol. The absorbance readings for samples were performed on a spectrophotometer (Multiskan Go, Thermo Scientific Brand).

CAT activity was measured using a catalase assay kit (code: ECAT-100), the detection range of the kit was 0.2–5 U/L CAT. This assay directly measures the catalase degradation of hydrogen peroxide (H_2_O_2_) using a redox dye. The change in colour intensity at 570 nm or fluorescence intensity is directly proportional to the catalase activity in the sample. The activity of SOD was measured using the superoxide Dismutase Assay Kit (code: ECAT-100); the detection range of the kit was 0.05–3 U/mL SOD. In the assay, the xanthine oxidase (XO) catalyzed reaction provides superoxide (O_2_-). O_2_- reacts with a WST-1 dye to form a coloured product. SOD removes O_2_- hence less O_2_- is available for the chromogenic reaction. The colour intensity at 440 nm is used to determine the SOD activity in a sample. In relation, GPX was measured using Glutathione Peroxidase Assay Kit GPX (code: EGPX-100); the detection range of kit was 40 to 800 U/L GPX activity. The assay directly measured nicotinamide adenine dinucleotide phosphate (NADPH) consumption in the enzyme coupled reactions. The measured decrease in optical density at 340 nm was directly proportional to the enzyme activity in the sample. Finally, GSH was measured using the Glutathione Assay Kit (code: DIGT-250; the detection range of kit was 0.4–100 μM. This improved 5,5′-dithiobis 2-nitrobenzoic acid (DTNB) method combines deproteination and detection (Reagent A) into one reagent. DTNB reacts with reduced glutathione to form a yellow product. The optical density, measured at 412 nm, is directly proportional to glutathione concentration in the sample.

### 2.5. Statistical Analysis

Data were tested for normality using the UNIVARIATE procedure (SAS Inst. Inc., Cary, NC, USA) with the normality of distributions determined by the Shapiro–Wilk (W) test. For the antioxidant enzyme variables, a completely randomized block design with nine replications per treatment was performed using the following model:

(3) $$Y_{ij}=\mu +T_{i}+B_{j}+e_{ij}$$

where *Y_ij_* is the observed value; *μ* is the overall average; *T_i_* is the fixed effect of the treatment; *B_j_* is the random block effect (animal); *e_ij_* is the random error associated with each observation.

For analyzing the pH, colour, lipid, and protein oxidation at a specific time, a split-plot design was used, employing the following model:

(4) $$Y_{ijk}=\mu +T_{i}+B_{j}+D_{k\;+}\;(T_{i}*D_{k\;})\;+e_{ijk}$$

where *Y_ijk_* is the observed value; *μ* is the overall average; $T_{i}$ is the fixed effect of the treatment; *D_k_* is the time in days;$\;(T_{i\;}$* $D_{k})$ is the interaction of the treatment and time; *B_j_* is the random effect (animal); *e_ijk_* is the random error associated with each observation. In addition, preslaughter live weight was used as a covariate. All data were evaluated using the MIXED procedure (version 9.4) from the statistical package (SAS Institute Inc., Cary, NC, USA, 2017). The level effect was evaluated using orthogonal polynomials, separating linear, quadratic, and cubic effects on physicochemical attributes and enzyme concentrations in meat. Because the intervals between treatments were not equidistant, the contrast coefficients were generated by the interactive matrix language (IML) procedure. Finally, the treatment averages were generated using the LSMEANS option (SAS Inst. Inc., Cary, NC, USA), and the averages were considered significant at *p* ≤ 0.05.

## 3. Results

### 3.1. pH and Colour Determination

The pH values were significantly influenced by exposure time (*p* = 0.007) (Table 2). However, dietary treatment (*p* = 0.5576) and the diet × time interaction (*p* = 0.087) did not affect the pH value. All colour variables in meat were significantly influenced by exposure time (L*, a*, b*, C*, and H*).

The intensity of yellowness of meat was significantly affected by the diet for treatments with 1 and 4% YME (*p* = 0.0035), where the treatment with 4% YME had the highest mean values for the d storage times (Figure S1) between the treatments analyzed, as can be seen in Table 2 (Figure S1). This effect was associated with the linear increase in the exposure time (*p* = 0.0012). Therefore, the averages of each treatment were used to generate a linear trend graph represented by the following equations:

y = 0.16x + 14.24 (R^2^ = 0.75) (5)

for the group with YME 1% and

y = 0.665x + 14.78 (R^2^ = 0.7949) (6)

for the group with 4% YME. A linear increase in C* was also observed over time of exposure for the treatment with 4% YME (*p* = 0.008) represented by the following equation (Figure S2):

y = 0.1x + 22.63 (R^2^ = 0.0092) (7)

### 3.2. Lipid and Protein Oxidation

The lipid oxidation values showed average values between 0.72 and 1.54 ± 0.09 mg of MDA per kg of meat, which increased linearly with increasing exposure time (*p* = 0.0001) (Figure S3). Moreover, dietary treatment (*p* = 0.3879) and the diet × time interaction (*p* = 0.9007) did not have a significant effect on this parameter (Table 2). For protein oxidation at retail display time (0, 3 and 6 days), there was no difference between treatments (*p* = 0.1027). However, the carbonyl values were affected by retail display time (*p* = 0.0256), which resulted in a linear effect (*p* = 0.0185) (Figure S4). Despite this effect, no significant variations were found in the diet × time interaction (*p* = 0.1498).

### 3.3. Antioxidant Enzymes 

Table 3 shows the results obtained in the quantification of antioxidant enzymes in the meat of lambs fed diets with the inclusion of YME. There were no statistically significant differences for the enzymatic activity of CAT, GSH, GPX, and SOD between the treatments analyzed (*p* ≤ 0.7891). The concentration GPX enzyme linearly increased (*p* = 0.0316) with the inclusion of YME. 

## 4. Discussion

### 4.1. pH and Colour Determination

The observed values were within the normal range (5.50 to 5.80) for sheep meat as previously reported [39,40,41,42], indicating adequate acidification of the meat for all the evaluated groups. These values were also similar to those found by Nieto et al. [15] and Yagoubi et al. [41], who used rosemary leaves as sheep feed and rosemary distillation residues in lamb diets, respectively.

The exposure time was expected to affect both the pH and colour of the meat. As the display time increases, the meat becomes more susceptible to oxidation and changes colour [43]. No significant differences in meat colour were found with the inclusion of YME in the diets, and the L* and a* values were within the normal ranges.

The lightness values, obtained for the evaluated groups, showed values that ranged between 41.61 and 45.41. Higher L* values result in lighter meat colour [44,45]. Regardless of the YME inclusion level, the meat of the animals used in this experiment was light. 

The b* parameter indicates the amount of carotenoid pigments of intra and intermuscular fat tissue [46]. Moreover, lipid concentration and the intake of carotenoid pigments contained in the green roughage can influence the yellow intensity of the meat and, consequently, the b* parameter [47]. The b* values in the evaluated treatments can be explained by the type of diet that the animals received because animals fed with diets containing grains and silage have low pH, which correlates negatively with the b* values [48,49]. In turn, the presence of secondary compounds in the extract may also have influenced the values in the 4% YME treatment, but no studies have reported the correlation of yerba mate supplementation with high values of yellow. Therefore, the observed increase in b* values may have been associated with oxidative processes, which produce Schiff pigments (lipofuscin) from lipid and protein complexes [50]. These pigments are positively related to the sensory appreciation of degradation of colour [51].

### 4.2. Lipid and Protein Oxidation

Lipid oxidation is related to meat discoloration [52], and the accumulation of carbonyl compounds caused by oxidation of unsaturated fatty acids and phospholipids in meat, which, in turn, is correlated with the oxidation of myoglobin in meat [53].

According to the study by Greene and Cumuze [54], who determined the relationship between TBARS values and inexperienced assessments of oxidized lipid taste in beef, a MDA range of 0.6–2.0 was necessary for panelists to detect oxidized flavors in the meat. In contrast, Campo et al. [55] reported that a MDA level of only 2.0 mg/kg tissue was required for consumers to be able to detect rancidity.

Ripoll, Joy, and Muñoz [56] stated that the acceptability threshold for oxidized meat varies according to the animal and the study, and an acceptability limit of 1 mg MDA/kg meat is more appropriate for lamb than the higher limits considered for beef. The results obtained in this study were low and within normal ranges, presenting values lower than or similar to those of other studies by Chikwanha et al. [57], Parvar et al. [58], and Salami et al. [59], who evaluated the use of natural products containing polyphenols.

The lack of effect of the YME treatment may be associated with the low pH values found in the present study. This translates into greater stability detected in the red colour parameter (a*) in all treatments at the three storage times, which is related to a delay in the oxidative process, generating less production of methemoglobin [15], as reflected in the TBARS content. Low TBARS values have also been positively associated with an increase in total polyunsaturated fatty acids (PUFA) (both *n*6 and *n*3) in the meat of lambs fed products rich in phenolic compounds [60].

Chemical modifications in the oxidative process include a loss of sulfhydryl groups, ketone formation, aldehyde formation, and amino acid oxidation, which depend largely on their position within the chain [61]. Certain amino acids, particularly cysteine, tyrosine, phenylalanine, tryptophan, histidine, proline, arginine, lysine, and methionine, quickly convert to carbonyl derivatives as they are relatively susceptible to reactive oxygen species [62]. In the exposure of amino acids to oxidation, carbonyl formation is considered a useful general indicator for assessing the level of protein oxidation in meat [63,64].

The observed values of carbonyls were considered high compared to those obtained by Chikwanha et al. [60], who evaluated different inclusion levels of grape pomace in lamb feed, and Santé-Lhoutellier et al. [64], who evaluated the effect of concentrate or pasture diet on the storage and oxidation of myofibrillar proteins.

Oxidative reactions can be easily transferred from lipids to proteins, showing a strong interaction between them [65]. Oxidizing lipids may react with proteins through free radicals and initiate protein oxidation [66]. This phenomenon may help explain the values obtained for the carbonyl and TBARS contents in the present study, showing a positive relationship between them.

As indicated by Estévez [65], measuring the carbonyl content does not allow for the evaluation of oxidative damage as a whole. Therefore, Mercier et al. [67] proposed the hypothesis that only certain amino acids produce carbonyl groups and that others, such as tyrosine and tryptophan, can be oxidized without producing carbonyl forms, demonstrating that the content of carbonyl compounds does not represent the full extent of the oxidation process.

However, more specific methods have been proposed. These methods are based on the detection of carbonyl proteins using Western blots, and γ-glutaminic semialdehyde (GGS) allows for the identification of individual oxidized proteins [68]. Carbonyl proteins are also considered adequate indicators of protein oxidation because they represent up to 60% of the total carbonyl compounds in food systems [69].

To date, threshold values for the carbonyl content in meat have not been established [60]. This area of study deserves further research because protein oxidation not only has negative effects on color and texture but also causes nutrient loss, including that of essential amino acids, and decreases digestibility [70].

### 4.3. Antioxidant Enzymes 

Analysis of antioxidant enzymes such as CAT, GPX, GSH and SOD are important because they constitute an endogenous intracellular barrier against free radicals, whose activity is modulated by various factors, such as stress, cell injury, or slaughter [71]. These enzymes act to transform reactive species into non-radical and non-toxic products [72].

Berté et al. [73], who evaluated the antioxidant activity of yerba mate, found that yerba mate has catalase-like activity, which is related to the total polyphenol content, but that the extract does not have superoxide dismutase activity. Therefore, catalase was expected to exhibit differences with various dietary YME inclusion levels in the present study. SOD and CAT are paired enzymes. SOD inactivates hydrogen peroxide, which is then converted by CAT into water and oxygen [67,74]. Thus, SOD and CAT should have similar values in their activity, but this was observed for only the control treatment. In the 1% YME treatment, the concentration of SOD increased as the value of CAT activity increased. 

GPX decomposes both hydrogen peroxide and lipoperoxides formed during lipid oxidation [75]. Consequently, the concentration would be expected to be associated with the oxidative process in meat. Considering that GPX is a selenodependent enzyme, which uses glutathione as a cofactor, the decrease in its concentration may be associated with the depletion of its cofactors, or the oxidation of proteins and lipids during retail display time, in turn, may indicate a deterioration in red and yellow in meat. According to the previous information, the increase in GPX activity in this research is related to the low TBARS values and greater stability registered in the colour of the lamb meat.

The lack of differences in enzyme activity with the use of YME may be explained by the fact that all cells enter anoxia and deplete their nutrients after the animals are euthanized and bled [71]. Under these conditions, enzyme activity can be considered representative only at the beginning of cell death. Research on antioxidant activity in meat shows different results [71]; in addition, the activity of some enzymes may differ from one species to another. Therefore, variations in enzyme activity between different genetic types can lead to differences in oxidative stabilization in meat [76]. However, these extracts exhibited antioxidant activity in vivo, which agreed with the study by Lobo et al. [32], who added yerba mate to lambs diets, reporting an improvement in antioxidant status.

## 5. Conclusions

Based on current findings, YME can be adopted as a food ingredient in 4% of lamb diets as a strategy to improve the visual appearance of meat, which is a factor that significantly influences consumer acceptance. Levels of up to 4% YME did not show negative effects on the quality traits in the meat, nor in the lipoperoxidation values during a retail display time of 6 days, maintaining the oxidative stability of the meat. In future studies, they should continue to evaluate the potential use of YME as an antioxidant in the quality traits of the resulting meat products, as there is little scientific information on this topic.

## Figures and Tables

**Table 1:** Ingredients and chemical composition of the experimental diets.

Component	Treatments			
	0%	1%	2%	4%
*Ingredients, % DM*				
Corn silage	40.00	40.00	40.00	40.00
Ground corn	33.00	33.00	33.00	33.00
Soybean meal	20.50	20.50	20.50	20.50
Salt	0.15	0.15	0.15	0.15
Dicalcium phosphate	0.15	0.15	0.15	0.15
Mineral Mix ^1^	2.20	2.20	2.20	2.20
YME	0.00	1.00	2.00	4.00
Kaolin ^2^	4.00	3.00	2.00	0.00
*Chemical composition*				
Dry matter (DM), % as-fed	68.89	68.83	68.77	68.64
Organic matter % DM	89.56	90.43	91.30	93.04
Crude protein % DM	21.46	21.56	21.66	21.87
Ether extract % DM	2.09	2.09	2.09	2.09
Ash % DM	13.77	10.85	8.47	7.16
Non-fiber carbohydrate % DM	51.90	52.69	53.49	55.07
Crude fiber % DM	13.87	13.88	13.88	13.88
ADF % DM	18.19	18.20	18.20	18.22
NDF % DM	34.53	34.54	34.54	34.56
Lignin % DM	4.48	4.48	4.48	4.48
Gross energy (kcal/kg)	4.19	4.23	4.27	4.35

^1^ Mineral Mix: GuabiPhos^®^, which has the following guaranteed levels per kilogram of product: calcium (maximum), 150 g; calcium (minimum), 130 g; phosphorus (minimum), 65 g; sodium (minimum), 130 g; fluorine (maximum), 650 mg; sulfur (minimum), 12 g; magnesium (minimum), 10 g; iron (minimum), 5000 mg; iodine (minimum), 60 mg; selenium (minimum), 10 mg; vitamin A (minimum), 50,000 international unit (IU); vitamin E (minimum), 312 IU. ^2^ Kaolin (also called the osp vehicle): kaolin is inert and has no nutritional value and is used to complete the ration.

**Table 2:** Effect of dietary treatment with yerba mate extract and retail display time (0, 3, and 6 days) on the physical and oxidative stability of lipids and proteins in the *Longissimus* *thoracis* muscle of lambs.

Item ^1^	Time 0				Time 3				Time 6				SEM ^2^	*p*-Value			
	0%	1%	2%	4%	0%	1%	2%	4%	0%	1%	2%	4%		Dieta	Tempo	D × T	Lineal
pH	5.57	5.66	5.40	5.60	5.66	5.66	5.67	5.8	5.66	5.63	5.68	5.62	0.06	0.5576	<0.0070	0.0873	0.6360
L*	42.77	41.61	42.42	43.20	45.16	43.20	44.30	45.41	42.69	43.09	42.05	44.55	0.86	0.1605	<0.0021	0.7204	0.1529
a*	16.80	17.29	16.92	17.30	14.75	14.20	14.60	14.80	15.03	15.39	16.24	16.25	0.50	0.5747	<0.0001	0.6614	0.1757
H*	40.36	39.72	40.76	42.20	47.04	45.00	47.20	46.77	45.65	44.93	44.30	46.25	0.92	0.3150	<0.0001	0.6266	0.2329
b*	14.26 ^a^	14.40 ^a^	14.57 ^a^	15.60 ^b^	15.77 ^a^	14.40 ^b^	15.70 ^a^	15.72 ^a^	15.31 ^a^	15.36 ^a^	15.75 ^a^	16.97 ^b^	0.37	0.0035	<0.0001	0.0997	0.0012
C*	22.05 ^a^	22.50 ^a^	22.34 ^a^	23.30 ^b^	21.62 ^a^	20.23 ^b^	21.50 ^a^	21.63 ^a^	21.50 ^a^	21.76 ^a^	22.69 ^a^	23.53 ^b^	0.52	0.0398	<0.0005	0.3069	0.0080
TBARS	0.80	0.79	0.74	0.72	1.02	0.94	0.86	0.93	1.43	1.54	1.35	1.34	0.09	0.3879	<0.0001	0.9007	0.1807
Carbonyl	22.36	23.18	28.22	17.20	22.96	29.61	39.97	66.22	24.98	20.99	33.38	33.29	8.90	0.1027	0.0256	0.1498	0.0185

^1^ L*: Lightness; a*: redness; b*: yellowness; C*: chroma; H*: angle hue; TBARS: mg malonaldehyde (MDA/kg); Carbonyls: nmol/mg protein. ^2^ SEM: standard error of the mean. ^a,b^ Means followed by distinct letters among treatments of each exposure time indicate significant differences (*p* ≤ 0.05).

**Table 3:** Effect of yerba mate extract (YME) supplementation on the antioxidant status of lambs.

Variables ^1^	Inclusion Level of YME				SEM ^2^	*p*-Value ^3^	
	0%	1%	2%	4%		Diet	Linear
CAT (U/L)	4.89	5.19	4.49	6.27	0.92	0.5643	0.3177
GPx (U/L)	103.68	146.44	193.78	341.84	83.88	0.1706	0.0316
GSH (μM)	60.58	67.05	66.44	60.91	6.00	0.7636	0.8741
SOD (U/mL)	0.06	0.07	0.06	0.05	0.01	0.7891	0.6573

^1^ CAT: Catalase, GPX: glutathione peroxidase, GSH: activity glutathione, SOD: superoxide dismutase. ^2^ SEM: standard error of the mean. ^3^ *p*-value: values from treatment (diet) and orthogonal contrast (linear) are significantly different if *p* ≤ 0.05.

## Supplementary Materials

The following are available online at https://www.mdpi.com/2076-2615/10/9/1458/s1, Figure S1: Effect of YME inclusion on parameter *b** during retail display time. ^a,b^ Means with distinct letters among treatments within each retail display time indicate significant differences in diet (*p* ≤ 0.05). ^x,y,z^ Means with distinct letters among the retail display times show significant differences (*p* ≤ 0.05); Figure S2: Effect of YME inclusion on values *C** during retail display time. ^a,b^ Means with distinct letters among treatments within each retail display time indicate significant differences in diet (*p* ≤ 0.05). ^x,y,z^ Means with distinct letters among the retail display times indicate significant differences (*p* ≤ 0.05); Figure S3: Effect of YME inclusion on the lipid oxidation values of *Longissimus thoracis* muscle at different retail display time expressed in mg malonaldehyde (MDA)/kg. ^x,y,z^ Distinct letters among the exposure times indicate significant differences (*p* ≤ 0.05); Figure S4: Effect of YME inclusion on the protein oxidation values of *Longissimus thoracis* muscle at different retail display times expressed in carbonyl (nmol/mg of protein). ^x,y,z^ Distinct letters among the exposure times indicate significant differences (*p* ≤ 0.05).
