# Supplementary material for: Effects of Feeding Increasing Levels of Yerba Mate on Lamb Meat Quality and Antioxidant Activity
Source: Animals (Basel). 2020 Aug 20;10(9):1458. doi: 10.3390/ani10091458 (PMC7552698; doi:10.3390/ani10091458)
Supplement: Supplementary file 1 [file animals-10-01458-s001.pdf]

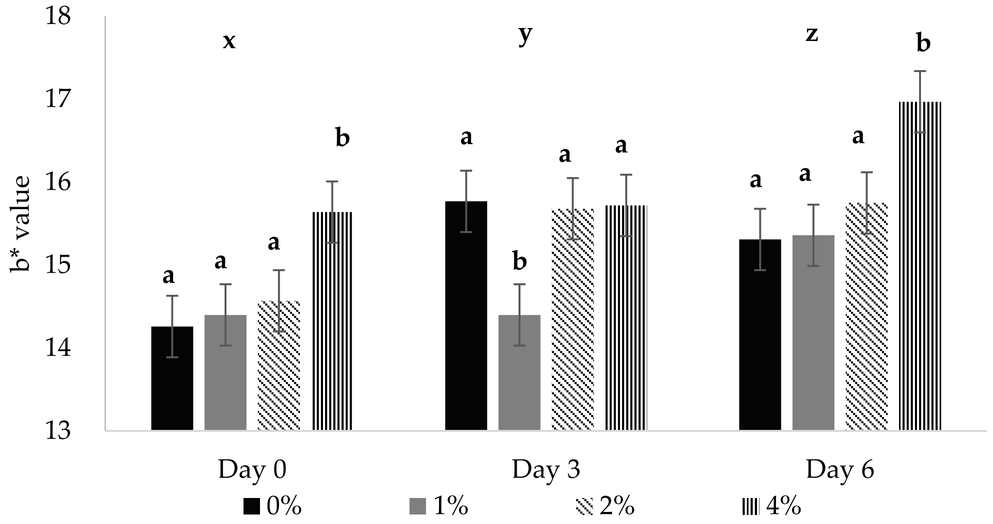

Figure S1. Effect of YME inclusion on parameter b* during retail display time. [a,b] means with distinct letters among treatments within each retail display time indicate significant differences in diet ($p \leq 0.05$). [x,y,z] means with distinct letters among the retail display times show significant differences ($p \leq 0.05$).

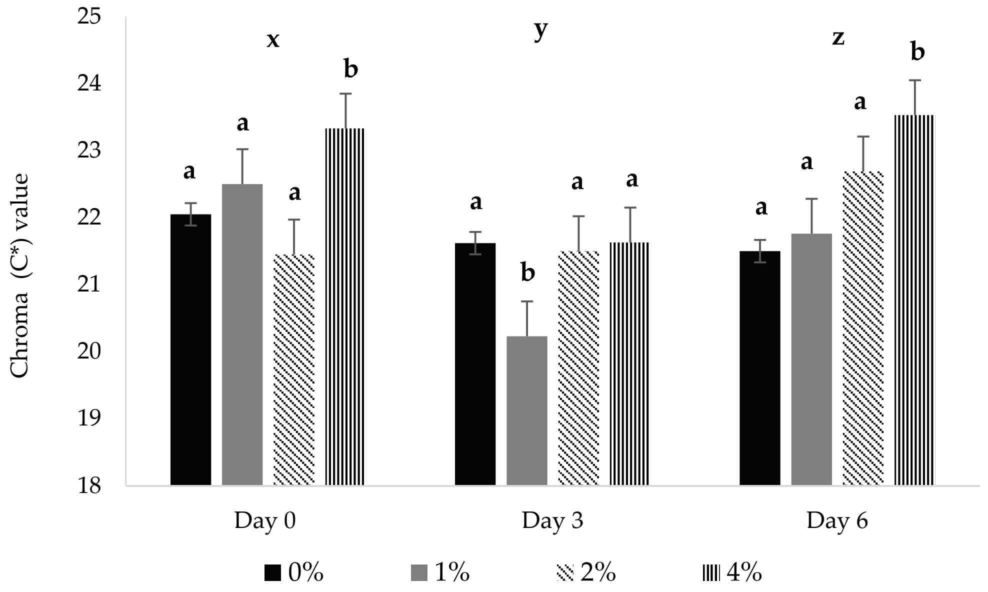

Figure S2. Effect of YME inclusion on values C* during retail display time. [a,b] means with distinct letters among treatments within each retail display time indicate significant differences in diet ($p \leq 0.05$). [x,y,z] means with distinct letters among the retail display times indicate significant differences ($p \leq 0.05$).

animals MDPI

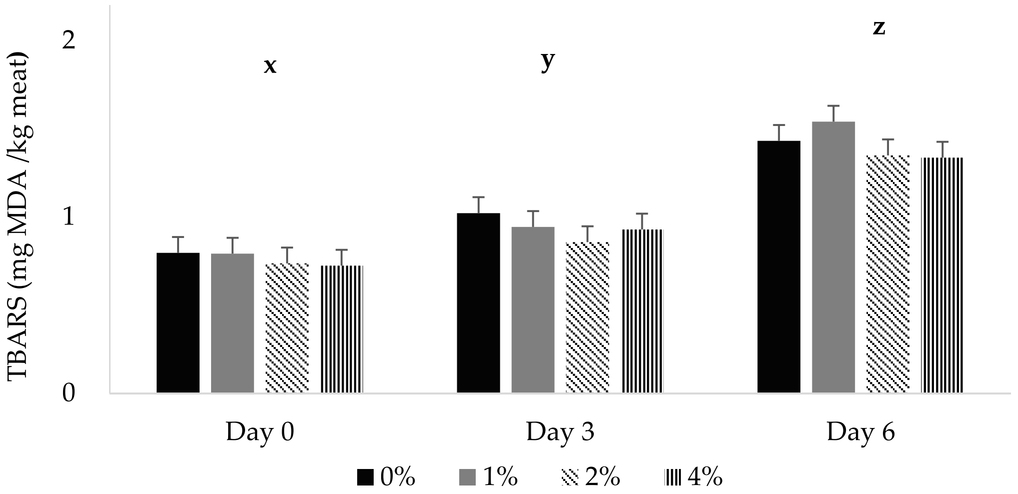

Figure S3. Effect of YME inclusion on the lipid oxidation values of Longissimus thoracis muscle at different retail display time expressed in mg malonaldehyde (MDA)/kg. [x,y,z] Distinct letters among the exposure times indicate significant differences ($p \leq 0.05$).

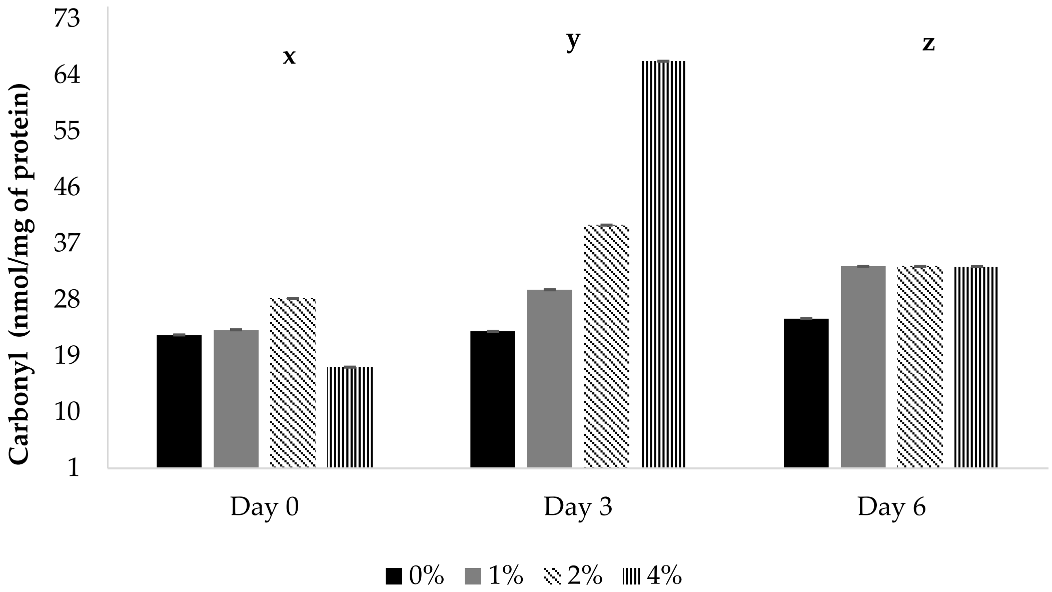

Figure S4. Effect of YME inclusion on the protein oxidation values of Longissimus thoracis muscle at different retail display times expressed in carbonyl (nmol/mg of protein). [x,y,z] Distinct letters among the exposure times indicate significant differences ($p \leq 0.05$).
